# Negotiating the ambiguity of an (in)authentic working life: a grounded theory study into severe perineal trauma

**DOI:** 10.1186/s12905-023-02191-9

**Published:** 2023-02-07

**Authors:** Katharina Tjernström, Inger Lindberg, Maria Wiklund, Margareta Persson

**Affiliations:** 1 Department of Nursing, Umeå University, Umeå, Sweden; 2 Department of Community Medicine and Rehabilitation, Section of Physiotherapy, Umeå University, Umeå, Sweden

**Keywords:** Severe perineal trauma, Maternal morbidity, Workability, Grounded theory, Gender theory, Simone de Beauvoir, Immanence, Transcendence

## Abstract

**Background:**

In Sweden, persistent physical and psychological health problems occur in about three in ten women who sustain severe perineal trauma (SPT) during childbirth. As most Swedish women work outside the home, the question of if and how SPT-related morbidity influences working life needs exploration. This study aims to qualitatively explore how women with persistent SPT-related morbidities experience and conceptualise their problems concerning working life. Further, we theorise the findings by applying Simone de Beauvoir’s feminist framework of immanence and transcendence as well as authentic and inauthentic life.

**Methods:**

Ten interviews with women recruited via a Swedish social media community for perineal trauma were analysed according to Charmaz’s constructivist approach to grounded theory.

**Results:**

The theoretical model and related core category ‘Negotiating the ambiguity of an (in)authentic working life’ reflected the women’s negotiations of immanence as ‘the silent covert object’ versus transcendence as the ‘the resourceful overt subject’. The model also mirrored the conflict of (in)authenticity in working life. An inauthentic working life was experienced when women were denied their subjectivity at work or constructed themselves as subjects in denial of their SPT. On the other hand, women who acknowledged their SPT and were constructed as subjects by ‘others’ achieved an authentic working life despite SPT.

**Conclusions:**

The conflicting gendered process of ‘the silent covert object’ versus ‘the resourceful overt subject’ problematised women’s vulnerability at work. Aspects that enable subjectification and transcendence are essential for policymakers, employers, healthcare services, and society to eradicate the taboo of SPT and create a working environment characterised by understanding, support, and flexibility. Further, access to adequate care, sick leave, and occupational rehabilitation are essential. Such measures support transcendence towards an authentic working life and, consequently, a more gender-equal working environment that does not deprive women of career opportunities due to a physical ailment.

## Background

### Introduction

Severe perineal trauma (SPT), i.e., third and fourth-degree perineal lacerations affecting the anal sphincter muscle and the anorectal mucosa [1], relates to short- and long-term (beyond one year postpartum) physical [2–6] and psychological [5, 7, 8] maternal morbidities. In Sweden, women experience more extensive STP-related morbidity than previously described [5, 8, 9]. Further, about one in three Swedish women report persisting SPT-related problems one year postpartum [9]. 78% of Swedish women work outside the home [10], and approximately 86% of Swedish one- to five-year-olds are enrolled in childcare [11]. This raises the question of if and how SPT influences women at work.

### Work-related consequences of genital problems and SPT

Globally, few studies explore how women with SPT, or other genital problems, experience their situation at work and whether the difficulties impact their professional role. Urinary incontinence, the most studied subtype of pelvic floor dysfunction, negatively impacts work productivity in men and women [12]. Experiencing urine incontinence at work manifests in reduced time management and diminished focus [13]. Women cope with urine incontinence at work by using pads, keeping spare clothing, limiting fluid intake, and frequently visiting the bathroom [14, 15]. Severe urine incontinence affects concentration, the performance of physical activities, self-confidence, or undisturbed task completion at work [14]. Faecal incontinence may lead to an inability to work part- or full-time, resulting in feelings of unfulfillment and a lack of work-related confidence. Employees with faecal incontinence frequently change pads and struggle with inaccessible toilets at work [16, 17]. Further, menopausal working women with uterine prolapse report worse occupational quality of life than mid-aged women without prolapse [18]. However, these studies do not explicitly study women with known SPT after childbirth.

The limited research on SPT-related problems in working life shows that women experience insecurities regarding incontinence at work [19]. On the other hand, working may distract their attention from SPT-related morbidities. Nevertheless, women may in some contexts lose their job due to anal incontinence after SPT [20]. In addition, they may need to change work tasks, occupations, or workplaces [19, 20], indicating that SPT-related morbidities may imply extensive professional consequences for some women.

### Gender perspectives on SPT

Gender norms and gendered power relations affect sexual and reproductive health within a social, economic, and political context [21]. Gender equality, sexual and reproductive health, and paid employment are part of the 2030 Agenda for Sustainable Development [22]. The lack of research on if and how morbidity after SPT influences workability suggests that women with SPT-related morbidity are an unrecognised and marginalised group despite being part of the workforce and contributing to society. Therefore, a gender perspective is essential to gain insight into these women’s working experiences. However, to our knowledge, such studies do not exist. A few studies use a feminist approach to explore perineal trauma, but with other perspectives. For example, Salmon [23] describes women’s experiences of exposure and vulnerability during suturing, the normalisation of postpartum pain by health professionals, and the complex emotional impact of perineal trauma, such as fear, anxiety, anger, frustration, and invisibility. Moreover, Priddis [24] shows that women with SPT use disembodiment strategies to disconnect themselves from their broken perineum and related problems to function in everyday life. Additionally, morbidity after SPT is found to cause blurring of boundaries between the ‘self’ and ‘other’ [25].

### Rationale

In sum, there is a global lack of research on the impact of long-term SPT-related morbidities on women’s working life, especially from a gender perspective. As most Swedish women work outside the home [10], the influence of persistent SPT-related morbidities on working life needs investigation. To address this knowledge gap, we aim to qualitatively explore how women with self-reported persistent SPT-related morbidities experience and conceptualise their problems concerning working life. Further, we theorised the findings by applying a theoretical feminist perspective.

### Theoretical and conceptual framework

To explore and theorise women’s ‘situated embodiment’ of SPT in working life, we apply concepts and notions developed by the existentialist feminist Simone de Beauvoir [26], see Table 1.

**Table 1:** Definitions of gender theoretical concepts according to Simone de Beauvoir [26]

Concept	Definition
Object	The body or nature
Subject	The mind
Situated embodiment	A lived incorporated experience in a particular situation and context
Immanence	Passive, repetitive, and monotonous existence as an object
Transcendence	Active existence as a subject pursuing developing life projects towards freedom
Ambiguity	Being object and subject at the same time
Authentic life	Embracing both immanence (the body) and transcendence (the mind) simultaneously
Inauthentic life	Denying the existence of either the body (immanence) or the mind (transcendence)
Authentic working life	As defined in the present study: Acknowledging one’s SPT-damaged body in working life while being constructed as a subject by others relevant to the work context
Inauthentic working life	As defined in the present study: Being denied subjectivity at work by others or constructing oneself as a subject in denial of the SPT-damaged body

Beauvoir’s well-known expression, ‘*One is not born, but rather becomes, woman*’ [26, p. 330], implies that ‘woman’ is a social and gendered construct in a specific situation and time. According to Beauvoir [26], ‘immanence’ represents the body or nature where the existence as an ‘object’ is maintained passively, repetitively, and monotonously. On the other hand, ‘transcendence’ symbolises the mind actively developing life projects towards freedom as a ‘subject’. Beauvoir sees the human being as ‘ambiguous’, incorporating both subject and object. To her, one can never deny one’s facticity, i.e., we are always a situated embodiment, a lived experience in a context. An individual can transcend to achieve an ‘authentic’ life from this immanent situation. Thus, according to Beauvoir, existing in only immanence or transcendence can constitute an ‘inauthentic’ life. She also argues that women are bound to face the conflict of freedom (transcendence) or reproduction (immanence) through their bodies and bodily processes, such as pregnancy and childbirth. These conflicts complicate transcendence: therefore, a woman’s body becomes an obstacle to freedom. For Beauvoir, women’s bodies are also the scene for constant body shame tied to sexuality and reproductive functions such as menstruation, sexual maturation, the postpartum body, or the development of breasts in puberty. Hence, ‘becoming a woman’ is a lesson in body shame. Thus, shame controls and oppresses women’s bodies regarding appearance and behaviour. Consequently, a woman cannot transcend due to her eternal status as an object and is therefore assigned to immanence.

Previously, Beauvoir’s ideas have been applied in research on sexual and reproductive health as well as working life. Studies on childbirth experience [27], motherhood [28], labour pain [29], and obstetric violence [30] problematise the gendered ambiguity embedded in these lived situations. Embodied ambiguity here embraces both possibilities (I can) and limitations (I cannot), subjectivity and materiality [29], independence and relationality [30], as well as stagnation and freedom [27]. Further, Beauvoir [26] views engagement in paid work outside the domestic field as a path of transcendence and freedom. Beauvoir [31] also critiques the gender-divided labour market where women are confined to certain femininely coded occupations with fewer career options. Veltman [32] states, using a Beauvoirian lens, that contemporary women are hindered in their transcendence because they are burdened with double work duty: employment outside the home and household chores. Further, Ross [33] analyses the professional role of nurses stating that the immanent values of ‘helping’ and ‘caring for others’ have been transformed into transcendent activities by present-day nurses.

Still, there is a need to theorise postpartum morbidity in various contexts. In the following, the Beauvoirian lens is applied to women with SPT and their working life as lived situation.

## Methods

### Study design

A constructivist approach to grounded theory (GT), according to Charmaz [34], inspired this study design. A GT of a studied topic starts with empirical data and ends with rendering them in an explanatory theory. Charmaz’s constructivist approach to GT also seeks to understand and explore complex social phenomena (such as working with SPT-related morbidities). Charmaz also stresses that the researcher is not a neutral observer but a co-participant in the study; thus, the findings are not discovered but constructed by the researcher and the study participants. During this construction process, we applied Simone de Beauvoir's theoretical framework [26].

### Study setting and Swedish context

A national sample of Swedish women with self-reported persistent SPT-related morbidities was recruited using the closed Swedish Facebook community ‘Förlossningsskadad? Du är inte ensam!’ (‘Injured at childbirth? You are not alone!’). This social media community addressing perineal trauma had over 7 600 members (during recruitment in 2020 to 2021), and only women with SPT can become members [35].

The employment rate of Swedish women (aged 20–64 years) was 78% in 2021, the highest rate in the European Union (EU) [36]. The healthcare (76.1%) and education sectors (71.1%) are dominated by women [37]. Sweden was estimated to have closed 82.3% of its overall gender gap by 2021, placing the country in the global top ten [38]. Women’s labour market outcomes decline after childbirth, salary and income decrease, and sick leave increases compared to men and pre-childbirth. This so-called child penalty effect may be explained by health status and gender norms [39]. The sickness rate is higher for Swedish women (26.35%) than men (17.78%) [36]. Swedish women’s sick leave at five years postpartum is mainly due to psychiatric diagnoses, such as depression or anxiety, and musculoskeletal diagnoses [39]. Since ICD-10 (International Statistical Classification of Diseases and Related Health Problems, 10^th^ Revision) diagnoses for late complications after perineal trauma were implemented as late as 2020 in Sweden [40], representative statistics on diagnoses and related sick leave are unavailable. The parental benefit consists of 480 days of paid leave (80% of annual gross wage) per child up to 12 years of age, and women are overrepresented in parental leave (70%) and leave to tend to sick children (60%) [36].

### Sampling and procedure

Purposive sampling was applied to aim for a heterogeneous sample of characteristics and SPT-related morbidities. The inclusion criteria were vaginal deliveries irrespective of mode, live-born child/children, third or fourth-degree perineal lacerations from January 2016 until June 2019, and self-reported persistent SPT-related morbidities. Further inclusion criteria were women above 18 years of age who understand and speak Swedish and have finalised the parental leave period (i.e., working, searching for work, studying or on sickness benefit). Exclusion criteria were stillbirths or death of an infant within 28 days, ongoing parental full-time leave after the injury, or subsequent ongoing pregnancy.

The community administrators posted a poster containing brief information about the study and a web link to the study homepage on November 23rd, 2020. Eligible participants could access further written information on the study homepage and contact the responsible researchers for additional information. Women who wished to participate notified the researchers and were contacted via telephone to ensure the fulfilment of inclusion criteria. According to the emergent study design, participants were included collaterally with interviews and preliminary analysis. A total of 12 participants contacted the research team during the first call. Interviews contained vivid, detailed descriptions of women’s experiences of working life and provided an extensive amount of data. Thus, our properties became analytically saturated when approaching ten interviews, providing the same concepts towards the end of data analysis. Therefore, there was no need to post another call for participants on the Facebook community. One woman emailed her interest to partake but did not respond to our contact efforts. One participant was excluded from this analysis as her problems did not relate to her working life. Final interviews were performed in February 2021.

### Data collection

The researchers developed a semi-structured interview guide focusing on working life. The construction of the interview guide was based on literature reviews, the sensitising concept of ‘gender’, and the clinical pre-understanding of the authors. KT, IL, and MP are midwives, and MW is a physiotherapist with expertise in gender theory. The interview guide was pilot tested by a woman who met the inclusion criteria except when the injury occurred and resulted in no significant changes. The individual interviews started with a broad, open-ended question concerning if and how SPT-related morbidity had affected working life. Additional questions covered physical, emotional, or social impact, management of symptoms, treatment by superiors or colleagues, sick leave, and thoughts about the future. Probing questions clarified and explored the concepts more profoundly.

Interviews were conducted via Zoom^®^ due to the Covid-19 pandemic and audio-recorded with the women’s consent. The interviews were carried out by the first author (KT), except for two interviews early in the data collection process, which IL or MW also attended. The interviews lasted 36–112 min with a median of 65 min and provided comprehensive data. Recordings were transcribed verbatim and validated for accuracy by listening to all recordings while reviewing the transcripts. The transcribed data were coded using MAXQDA^®^ [41], a software for analysing qualitative research data.

### Data analysis

In line with Charmaz [34], the coding process was initially inductive, followed by an abductive stage in which we applied the gender theoretical framework of Simone de Beauvoir [26]. The analysis started collaterally with the data collection, and the authors repeatedly discussed the emerging findings. Memos were written after each interview and during the analysis. All authors coded one of the first interviews, and the emerging initial codes were triangulated. Following Charmaz [34], KT inductively coded the remaining interviews naming lines or segments of data with predominantly longer in vivo and in vitro codes close to the data trying not to lose nuances. Verbs and gerunds (i.e., verbs ending with ‘-ing’) in initial codes aided in exploring inferred meanings and actions in the data—sparking interest to investigate different emergent links by constantly comparing data and going back to the text.

After the inductive initial coding process, the codes were clustered and visualised to identify the first central concepts during focused coding. Further, memos and the theoretical framework supported the construction of categories and gave ideas on theoretical constructs. Finally, theoretical coding identified how our categories related to each other. Abduction, memo-writing, and visual reflections aided this process. Analytical saturation was reached when the same concepts kept emerging, and after ten interviews, no new significant concepts contributed to our properties.

More specifically, during the theoretically informed phase of abduction, several gender theoretical perspectives were discussed to understand the meaning of data (e.g., intersectionality, crip theory or corporeal feminism). Since the emerging categories and social process pointed towards an equivocal, double-sided, and conflicting situation of passivity/stagnation (body) vs activity/development (mind) in working life, Simone de Beauvoir’s notions on immanence and transcendence [26] were adopted (see Table 1). This contributed to understanding the phenomenon at an abstract level and aided in further conceptualising and developing a theoretical model grounded in the emerging data. The emergent theoretical model was also peer-reviewed and discussed at research seminars.

### Ethical considerations

The research project was performed in accordance with the Declaration of Helsinki [42] and approved by the Swedish Ethical Review Authority (Dnr: 2020-035410908). Participation was voluntary, and the women could withdraw without stating a reason. Informed consent was mandatory and was obtained digitally before each interview. The women could refrain from answering questions they felt were too intimate. The project homepage and the information letter provided information on available support options. If any woman experienced negative emotions after the interview, researchers could assist in referral to adequate healthcare services.

## Results

### Participants

Background characteristics are found in Table 2. Participants were all cis women, i.e., identifying themselves as women, and subsequently referred to as ‘the women’ in this paper. The median age was 35.5 years (min.–max.: 25–42 years) and the mean number of children was 2 children (min.–max.: 1–4 children). All women were married or cohabited with their partners. The self-reported morbidities varied from urine or faecal incontinence, pain, sexual dysfunction, defecation problems, and prolapse to psychological symptoms, and most women reported a combination of morbidities. Most women had partial parental leave (an option within the social security system to reduce working hours for parents with young children). Women with no sick leave periods related to their SPT reported wishing they had been offered the possibility.

**Table 2:** Group-level demographic background data of 10 included participants

Demographic category	Number (n)
Country of origin	Sweden (7); Estonia (1); Syria (1); South Korea (1)
Degree of perineal laceration	Third-degree (7); Fourth-degree (3)
Year of SPT	2016 (4); 2017 (5); 2019 (1)
Education level	University level (7); Higher Secondary (2); Lower secondary (1)
Type of occupation	Physically challenging (5); Sedentary (5)
Type of employment	Permanent (7); Substitute (1); Unemployed (2); Student (2)
Employment rate	Full-time (4); Part-time (6) [20 to 83% of full-time]
Reason for sick leave	Reconstructive surgery (5); Other reasons than SPT (4)

### Negotiating the ambiguity of an (in)authentic working life

**Fig. 1:**
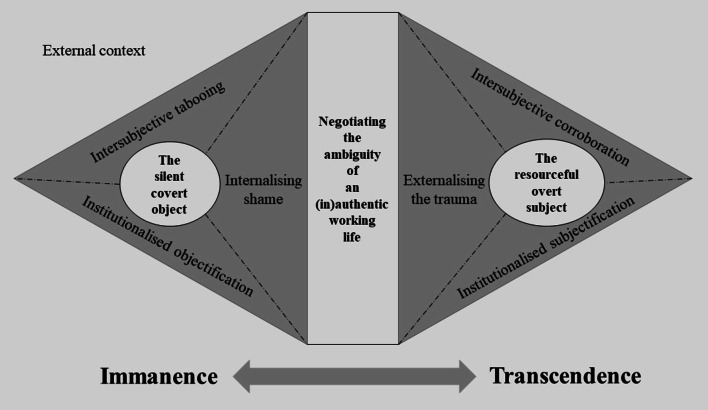
The theoretical model on women’s negotiations regarding the ambiguity of an (in)authentic working life

When returning to working life, the persisting consequences and traumatised bodies were the women’s shared experience and mutual departure as professionals. However, their return to work was multifaceted and discrepant, forming the base for our grounded theory of their situated embodiment. The theoretical model, inspired by Beauvoir [26], and the related core category ‘Negotiating the ambiguity of an (in)authentic working life’ reflected their negotiations of the conflicting gendered process of immanence versus transcendence at work (Fig. 1). The women negotiated work immanence as ‘the silent covert object’ by objectification and professional stagnation due to SPT. However, they could also experience work transcendence as ‘the resourceful overt subject’ who gained professional competency and freedom from restrictions at work despite SPT.

The interpretation of ‘ambiguity’ as the core of the traumatised professionals’ situated embodiment and professional ‘becoming’ refers to the women’s negotiations and efforts to be object and subject simultaneously. Specific individual, organisational, and institutional aspects played a role in this conflicting gendered process (Table 3). For instance, the women could be constructed or construct themselves as ‘transcending subjects’ at the individual and interpersonal level at work, e.g., by being open about their SPT and receiving support. Nevertheless, they were simultaneously objectified at the institutional level because healthcare services denied them adequate help and sick leave. This conflicting gendered process highlighted the vulnerability of working women with SPT-related morbidities.

**Table 3:** Overview of core category, categories, subcategories, and properties

Core category	Negotiating the ambiguity of an (in)authentic working life				
**Category**	**Immanence at the workplace—the silent covert object**		**Versus**	**Transcendence at the workplace—the resourceful overt subject**	
**Subcategory**	Internalising shame		**Versus**	Externalising the trauma	
**Properties**	Hiding the SPT	Shame as a restrictor		Being open about SPT	Being free of shame
**Subcategory**	Intersubjective tabooing		**Versus**	Intersubjective corroboration	
**Properties**	The restricting workplace	Tabooing SPT at work		Needing flexibility	Receiving support and understanding
**Subcategory**	Institutionalised objectification		**Versus**	Institutionalised subjectification	
**Properties**	Seeing women as objects	Struggling endlessly to get sick leave		Finally receiving care	Seeing women as subjects

Further, inauthenticity at work was reflected in the denial of subjectivity by objectification via internal or external ‘others’. An inauthentic working life was also experienced when women constructed themselves as subjects in denial of their facticity, i.e., a body with SPT. On the other hand, the women who gained access to their subjectivity through openness, support, and subjectification by ‘others’ achieved an authentic working life while simultaneously embracing their bodies with SPT.

The theoretical model should be understood in the context of gendered societal norms visualised as an external context consisting of society, workplaces, healthcare institutions, and national expert bodies, such as the social security agency or policymakers.

### Immanence and transcendence at the workplace–being ‘the silent covert object’ versus ‘the resourceful overt subject’

The (in)authentic working life comprised the negotiation of the ambiguity of being ‘a silent covert object’ and ‘a resourceful overt subject’, which took place at an individual, organisational, and institutional level.

Becoming ‘a silent covert object’ represented a gendered construct characterised by immanence and objectification at the workplace. The objectification occurred at different levels; ‘internalising shame’ at an individual level, ‘intersubjective tabooing’ at an organisational level, and ‘institutionalised objectification’ at an institutional level. Being seen as an object made women with SPT-related morbidities hide their condition because SPT was taboo in the workplace. Moreover, objectified women perceived themselves as ignored by healthcare services and, thus, received inadequate care. This lack of care contributed to stagnation at work because no rehabilitating measures were available to improve the work situation. Thus, the women were confined to embodying ‘the silent covert object’. Their ‘bodies’ were problematic in this gendered construct, and their resources as ‘minds’ were silent. Objectification was not a fixed status but ‘ambiguous’ and opened for negotiation as the women could embody both subject and object at different levels or in different work situations. They could define themselves as subjects but be objectified by others, or vice versa. The women experienced limited career options because they, for example, had to quit their jobs, put their studies on hold, and could not work as intended. Further, the women described socio-economic limitations. They used parental social security benefits to compensate for a default sick leave or reduced work time without compensation.

In contrast, the gendered process of becoming ‘a resourceful overt subject’ represents a gendered construct characterised by transcendence at the workplace despite the sequelae of SPT. Subjectification was portrayed by ‘externalising the trauma’ at an individual level, ‘intersubjective corroboration’ at an organisational level, and ‘institutionalised subjectification’ at an institutional level. To be understood and constructed as a subject conveyed the possibility of revealing the SPT, receiving support or needed aids at the workplace, and being seen as an individual in need of care and sick leave by societal institutions. The construct of women with SPT-related morbidities as subjects was crucial to experiencing transcendence at work. Thus, the women could break free from restrictions at work due to SPT and embodied ‘a resourceful overt subject’. Although constructing themselves as a working subject, some women faced ambiguous situations where the workplace or healthcare services did not recognise their subjectivity.

The ambiguity of an (in)authentic working life and being ‘a silent covert object’ and ‘a resourceful overt subject’ entailed negotiations, i.e., ‘internalising shame’ versus ‘externalising the trauma’ (individual level), ‘intersubjective tabooing’ versus ‘intersubjective corroboration’ (organisational level), and ‘institutionalised objectification’ versus ‘institutionalised subjectification’ (institutional level). In the following, we present the negotiations in more detail with citations from the interviews.

#### ‘Internalising shame versus externalising the trauma’

At an individual level, the women negotiated to internalise the shame of SPT versus externalising the trauma when facing the ambiguity of an (in)authentic working life.

Embodying ‘a silent covert object’ meant hiding their condition at work, and shame reinforced this process. Internalised shame implied that the women established a workplace behaviour characterised by adjustment, isolation, avoidance, or prevention, which interfered with their workability. The concealing behaviour was interpreted as passivity, stagnation, and resignation, corresponding to immanence. Thus, the women became an object; the women were ashamed of their SPT-related morbidities, and the workplace situation reinforced this feeling.

After all, it [incontinence] becomes some sort of shame. It does not fit the norm to be just over 30 and need a diaper. (Interview 8)

Shame restricted them from telling others at the workplace, asking for help, seeking medical help or sick leave. Some women used parental leave to cover up for diminished work capacity or came up with excuses for not participating in certain activities. Further, they were afraid that the SPT-related morbidities might damage their professional reputation and negatively affect how colleagues viewed them as professionals. As a result, they internalised others’ gaze and patronising in their self-esteem:

They [my colleagues] might think: Well, what does this [SPT] imply? Is this a person who maybe does not come to work because she must sit on the toilet at home? (Interview 3)

Some women adjusted their workplace behaviour by varying work tasks, working fewer hours, or performing more manageable tasks on a ‘bad day’. Moreover, the women isolated themselves professionally. This reduced work travels and colleague-related stress, gave easy access to a toilet or shower after bowel accidents, and facilitated alternating postures to relieve their aching bodies. Avoidance manifested in some women not seeking occupational healthcare and, therefore, denying their trauma. The women also avoided exposed situations to hide their condition from colleagues, such as afternoon meetings for fear of incontinence after lunch or work travels due to limited access to toilets. The women described how they avoided larger groups, heavy lifting, or high-stress levels because they feared public incontinence. Some women postponed visits to the bathroom to ensure privacy and preferred visiting the toilet at home.

I partly do not want them [my colleagues] to notice that it [toilet visit] takes some time. Moreover, in no way do I want to push [because my symptoms may worsen]. And when I am stressed, I may push. So, I must make the body feel calm to relax because it [faeces] then comes easier without pushing. (Interview 10)

The women revealed preventative measures to conceal their SPT, e.g., emptying their bowels before work, adjusting their nutrition, and wearing incontinence pads. They preferred digital meetings or worked effectively to cope and cover for a potentially bad period that might strain them. The women always ensured they had quick access to a toilet. A sense of security was established by knowing where the nearest toilet was. Unfortunately, locating the closest bathroom became an obsession for some women who internalised the shame attached to public incontinence.

But I am very thorough with knowing where a toilet is [located]. When I walk down the corridor, I know which direction to turn. Do I have to go one floor up or down? Do I have to ride the elevator? Do I have access to the elevator? (Interview 5)

In contrast, constructing themselves as subjects by being open about the SPT at work was a prerequisite to transcendence. It seemed crucial for the women to be honest about their SPT to become subjects at an individual level. Nevertheless, revealing the SPT was a long process where the women seemed to become more confident in sharing their SPT-related morbidities the longer they had been dealing with them. The women noted that being honest about their situation could be therapeutic, and they thought their revealing process could empower others to tell. Thus, these women embodied a subject where shame did not play a significant part. Women conceptualising themselves as subjects also listened to their needs and revealed positive thinking and hope.

So, I am frank; I have told them [my colleagues] everything. I perceive them to be really great and understanding. (Interview 7)

#### ‘Intersubjective tabooing versus intersubjective corroboration’

The women balanced between an intersubjective taboo where SPT was silenced at work and, on the other hand, an intersubjective corroboration consisting of understanding, support, and flexibility at work.

Objectification and detention in immanence were done both ‘by’ the women and ‘to’ the women. Intersubjective tabooing showed how the workplace objectified women by treating SPT as a taboo with restricted openness. Some women perceived their workplace as closed and intolerant, so they remained silent about their situation.

Well, it feels like you try to hide it as much as possible. It is not like saying, ‘I have diabetes, so if I collapse, give me a shot’. It does not feel like such a thing. (Interview 6)

Hence, they were met with incomprehension and a lack of support. Moreover, a restricting workplace made the women feel that SPT-related problems should only be mentioned if necessary. Consequently, many women did not tell their superiors or colleagues about their SPT, and if they shared their SPT, they did not disclose any details or only told certain people.

No, not my boss, […] because he would have no sympathies. I have only told him that I do not feel well and have complications. But I have not told him why. I was not comfortable telling my boss. (Interview 8)

The women experienced SPT as a gendered taboo in the working environment; being an injured or incontinent working young woman was not the norm. This gendered taboo also functioned as a barrier to being open about SPT at work. Most women felt uncomfortable telling men about the injury and did not feel understood by male co-workers.

It works both ways. He [the boss] is an older man, nearly 70 years old. Furthermore, he generally has minimal sympathy for women, and it was no idea to discuss this with him. So, it was both his personality, and he was male. I think a man cannot understand in the same way [as a woman]. (Interview 8)

Although SPT was perceived as taboo, some women felt forced to disclose their injury to get support or adjustments at work before reconstructive surgery or to explain any deviant behaviour.

In contrast, Beauvoir [26] suggests that a woman is not solely responsible for her status as an object or subject. Thus, intersubjective corroboration portrayed the women’s need for specific aids at work to take an active role as a working subject and functioning at work. The women described that flexibility during the workday was essential to work despite SPT-related morbidities; they needed to plan their workday, making them feel competent and able-bodied.

My work is relatively flexible, so I can slip away for a little while if I want to. I think it is worse to have a four-hour shift at the register at the supermarket because then you are supposed to sit there. (Interview 10)

Openness about SPT decreased stress levels and created a sense of security and relief, enabling the women to receive support and understanding. As a result, colleagues could take care of specific work tasks. Nevertheless, the women needed genuine interest and trust to reveal their SPT at work. Consequently, the women could access understanding, support, and flexibility, enabling them to embody working subjects.

I remember when we were supposed to go to xxx, we were supposed to sit in the car for two hours. So, I said, ‘Well, I decline’. I did not want to sit in that car for two hours with other people and not know where to use the bathroom during the car trip. So, I discussed this with my boss, and I did not have to go; I could decide if I wanted to join. So, I chose to stay and work instead. (Interview 5)

#### ‘Institutionalised objectification versus institutionalised subjectification’

At the institutional level, the women faced both objectification and subjectification by healthcare services or the social security agency in their negotiations of the ambiguity of an (in)authentic working life.

The institutionalised objectification manifested in healthcare providers’ perceptions of women and their genitals as objects and ignoring their healthcare needs. This lack of care left the women struggling to receive appropriate help and sick leave if needed. Consequently, a neglected diagnosis, inadequate support, and denial of sick leave enforced the women’s immanence at work. Some women experienced that the healthcare professionals reduced them to incorporating genitals when asking for help. Some women noted that SPT-related morbidities were taboo in healthcare services rendering these women voiceless because nobody was listening. Healthcare providers made them believe that symptoms were imagined by calling persistent symptoms ‘normal’ or a ‘psychiatric condition’. Some women only received sick leave for mental issues, which was perceived as gendered. Such objectification left the women feeling restricted at work and depressed due to a physical ailment. A denial of sick leave bound the women to incorporate ‘a silent covert object’ at work. Lack of care led to internalised acceptance; the health problems became normality, and the women were forced to ignore their symptoms to manage.

My pain level is so incredibly high that I do not remember what it was like not to be in pain. So, as every day goes by, I endure more and more pain. […] Well, it really hurts, but I have gotten used to it. Everyday life. (Interview 1)

In contrast, acknowledgement as a subject was crucial for transcendence. It was mirrored in the women’s experiences of subjectification by healthcare providers and the social security agency, which enabled access to medical care and sick leave. For example, some women described how healthcare providers acknowledged them and saw them as ‘actual patients with real symptoms’ needing treatment and sick leave.

I was so lucky to get hold of a physician who went totally nuts [finding out about my situation]. So, she has helped me to get help. So that physician, all credit to her, I have had so much help and support from her. She did not let go of me. (Interview 8)

The extent of their SPT was finally discovered if they met a healthcare provider who listened to them. Symptoms were taken seriously, which was experienced as a confirmation of not being mentally ill or imagining problems. Thus, the women appreciated an explanation of their difficulties and adequate help and support. This resulted in liberating feelings and validation of their subjectivity, which enabled transcendence in working life.

So, to be taken for real, to be listened to as a woman and not only reject women’s problems regarding birth injuries. (Interview 5)

In summary, working life for women with SPT was experienced as ambiguous and (in)authentic. The women negotiated to embody ‘the silent covert object’ and ‘the resourceful overt subject’, leading to a conflicting gendered process when they returned to work. The women’s experiences led them to perceive themselves as objects contained in immanence and subjects transcending any restrictions at work.

## Discussion

Our generated theoretical model, in which we applied the ideas of Simone de Beauvoir [26], demonstrated how the women with persistent SPT-related morbidities negotiated an (in)authentic working life and faced ambiguity regarding being object and subject. This entailed a negotiation of being objectified and doomed to ‘the silent overt object’ or subjectively constituted to be a ‘resourceful overt subject’. The conflicting gendered process of either becoming ‘the silent covert object’ or ‘the resourceful overt subject’ at work highlighted the vulnerability of the affected women, tied to inauthentic working life. In turn, authenticity referred to an integration of these constructs.

In terms of immanence and/or transcendence, our model illuminated an ambiguity regarding the situated embodiment of women with SPT at work. This contrasts with Beauvoir [26], who understands women’s bodily processes as solely immanent projects (in a patriarchal context). Therefore, our study may contribute significant insight into the complexity of SPT and differing working conditions. Likewise, postmodern feminist theorists [43] disregard Cartesian dualism and argue for an indivisible connection between the corporeal and the culturally constructed body. Our conceptualisation of ambiguity might also be compared with prior research on childbirth experience [27] and motherhood [28], concluding that childbirth and motherhood in terms of immanence/transcendence might be ambiguous experiences. Childbirth [27] both limits women’s freedom in life (immanence) and is interpreted as a meaningful and developing project for future life (transcendence). Further, motherhood [28], i.e., pregnancy, birthing, breastfeeding, and raising children, can be viewed as an ambiguous concept: both repetitive (immanence) and creative, joyful as well as empowering (transcendence).

From a Beauvoirian perspective [26], paid work is a way towards women’s transcendence. Based on our conceptualisation, this calls for embracing one’s SPT facticity at work and being subject and object in symbiotic ambiguity to achieve an authentic working life. Enhanced workability also requires a supportive workplace and organisation. As reflected in our study, findings from previous studies show that women with SPT may need to change work tasks, occupations, or workplaces [19, 20]. Further, SPT has been found to reduce work capacity [7], which was also visualised in our results; women developed an individualised workplace behaviour consisting of adjustment, isolation, avoidance, or prevention with a negative impact on their work capacity. The women in our study even used parental leave to cover up their reduced workability. Further, in previous research, women experience insecurities regarding incontinence at work [19], similar to our participants, who described how internalised shame made them hide their condition. Other studies have also found institutionalised objectification [44–46], where healthcare professionals normalise persistent morbidities or question the legitimacy of symptoms. The long struggle for adequate healthcare depicted in our study is also supported by others [25, 44, 47]. Hence, previous research shows almost solely adverse effects of SPT on working life. Only one study by Keighley et al. [20] describes that work may function as a distraction from SPT-related anal incontinence. Our theoretical model suggested multiple aspects of transcendence concerning working life. Externalising the trauma, intersubjective corroboration, and institutional subjectification were positive inclinations supporting the working women with SPT in our study. Thus, they could function at work despite SPT. As mentioned, the research on SPT-related morbidities at work is limited [19, 20]. Our findings corroborated these few studies but may also deepen the insights into the occupational situation of women with SPT.

Our conceptualisation of internalised shame indicated that a body with SPT-related morbidities was not congruent with existing norms of corporal occupational femininity and therefore silenced at work. Shame [48] is a lived experience and is consequently embodied. There must be an audience and ‘gaze’, internal or external, for shame to occur. Shame can therefore be experienced due to an internal self-devaluation process where the subject judges itself through an internalised other, e.g., a ‘male gaze’ or a ‘biomedical gaze’. These ideas align with our findings, where shame seemed to urge an internalisation process of hiding SPT at work. Such concealments of health conditions at work [49, 50] are documented for other illnesses such as migraine, fibromyalgia, or multiple sclerosis. In our study, the women struggled to disclose SPT at work, negotiating the ambiguity of ‘internalising shame’ and ‘externalising the trauma’. Similar ambiguity and dilemmas are presented in other studies [51, 52] where workers struggle with disclosing chronic illnesses at work. Further, menstruation [53], pregnancy [54], and breastfeeding [55] are reproductive processes depicted as taboo in the workplace. Herron-Marx, Williams and Hicks [45] show that perineal morbidity is a taboo for women themselves and society. Moreover, Keighley et al. [20] describe the hidden taboo of living with SPT. These study findings cohere with our conceptualisation of SPT as an objectifying taboo at the workplace and a demonstration of power by shaming working women with deviant bodies.

Some women in our findings used partial parental leave to cover up their impaired workability. This might misrepresent women’s need for work adjustments or sick leave absence and underestimate their health status postpartum. Generally, pregnancy and childbirth cause no new medical diagnoses requiring sick leave, but sick leave periods increase after childbirth [39]. Underlying health problems, more common among women with lower socio-economic status, can be reinforced after childbirth [39]. Postpartum morbidity can then be disguised by parental leave. Our study highlighted that childbirth might imply new health problems that negatively influenced the women’s work situation and might delay professional transcendence and career. In a wider Beauvoirian sense [26], women with SPT do not only strive towards transcendence in regard of freedom from impaired workability due to their SPT but also in regard of economic freedom after childbirth. Working only part-time or compensating default sick leave with parental benefit leads to lesser income and pension compared to their partners; an economical gap that women globally are already trying to close [22, 38]. This financial loss places women with SPT back in financial dependence on their partner; an ‘oppression’ that paid work itself should have freed them from according to Beauvoir [26].

### Strengths and limitations

Charmaz [34] speaks of credibility, originality, resonance, and usefulness regarding trustworthiness.

The transparent description of our methodology, a detailed description of our categories, including extensive and vivid descriptions of various experiences, and interdisciplinary authors’ triangulation enhanced credibility [34]. Our preunderstanding, a part of credibility [34], origins from the clinical expertise of midwifery or physiotherapy and research addressing perineal trauma and gender studies. We identify as women, feminists, and mothers. The positive aspects may include a vast clinical experience of the subject, and some intimate questions may be easier to discuss with a female researcher. On the other hand, our preunderstanding of the problems may bias our perceptions. However, our different professional backgrounds and repeated triangulation within the research group and in seminars may have minimised any possible preunderstanding bias. In line with Charmaz [34], we also practised reflexivity by being aware of our preunderstanding, using it to construct our theoretical model, and routinely writing memos on analytical choices.

Further, our study has originality [34] because we contribute novel findings and a theory grounded in data to an area that, to our knowledge, has not been researched before.

Thus, our study conveys new insights and concepts that are socially and theoretically significant since they may prove helpful to policymakers, employers, and healthcare services [34]. Further, the findings, depicted with thick descriptions, reflect the experiences of our sample and might be transferable to other women with SPT in similar working contexts, adding to the usefulness of the paper.

When saturation is obtained is a subject of debate; some suggest about 12 participants [56], while others argue for up to 20 participants [34]. This paper could have had a larger sample by repeating the call for participants. However, the ten included interviews contained vivid, detailed descriptions providing extensive data, and the same concepts reappeared towards the end of the analysis. Thus, sufficient information power [57] was obtained, and the researchers are confident that the constructed theoretical model was grounded in data that may resonate with the whole picture. In addition, three women were born outside of Sweden, which adds to resonance [34] and is a strength as immigrant women are often excluded from research. Our study also included women with both sedentary work and physically demanding occupations, which further strengthens the resonance of the paper. Nevertheless, future studies should address the possible differences in experiences depending on the type of occupation. Our sample included only cis women in relationships, which may not reflect the experiences of same-sex parents or single parents. Moreover, most women had higher education and the majority had third-degree lacerations. This does not fully reflect the experiences of women with fourth-degree lacerations or those with lower levels of education. Our results also included more data on immanence than transcendence. These aspects impact the paper’s resonance, and future studies should focus on these groups.

## Conclusions

We showed that women with persistent SPT-related morbidities after childbirth experienced an ambiguity of (in)authentic working life. The women had to constantly negotiate being objectified and doomed to ‘the silent overt object’ or subjectively constituted to be a ‘resourceful overt subject’. Creating a working environment characterised by understanding, support, and flexibility is essential to eradicate the taboo of SPT-related morbidities in working life. Thus, policymakers, employers, healthcare services, and society should focus on enabling subjectification and transcendence in working life. Therefore, further research is needed to develop an occupational rehabilitation programme focusing on SPT-related morbidities, which, to our knowledge, does not exist. Furthermore, healthcare services, employers, and social security agencies should collaborate to provide adequate medical help and support so that women can access social security resources when needed. Such measures can support transcendence towards an authentic working life and a gender-equal working environment that does not deprive women of career opportunities due to a physical ailment.

## Data Availability

The datasets generated and analysed during the current study are not publicly available due to informed consent and confidentiality but are available from the corresponding author upon reasonable request.

## Abbreviations

EU: European Union
GT: Grounded theory
ICD-10: International statistical classification of diseases and related health problems, 10th revision
SFO-V: Strategic Research Area Health Care Science
SPT: Severe perineal trauma
